# An instrumented centrifuge for studying mouse locomotion and behaviour under hypergravity

**DOI:** 10.1242/bio.043018

**Published:** 2019-06-12

**Authors:** Benjamin J. H. Smith, James R. Usherwood

**Affiliations:** Structure and Motion Laboratory, Royal Veterinary College, Hawkshead Lane, Hatfield, Hertfordshire AL9 7TA, UK

**Keywords:** Locomotion, Hypergravity, Biomechanics, Automated measurement

## Abstract

Gravity may influence multiple aspects of legged locomotion, from the periods of limbs moving as pendulums to the muscle forces required to support the body. We present a system for exposing mice to hypergravity using a centrifuge and studying their locomotion and activity during exposure. Centrifuge-induced hypergravity has the advantages that it both allows animals to move freely, and it affects both body and limbs. The centrifuge can impose two levels of hypergravity concurrently, using two sets of arms of different lengths, each carrying a mouse cage outfitted with a force and speed measuring exercise wheel and an infrared high-speed camera; both triggered automatically when a mouse begins running on the wheel. Welfare is monitored using infrared cameras. As well as detailing the design of the centrifuge and instrumentation, we present example data from mice exposed to multiple levels of hypergravity and details of how they acclimatized to hypergravity.

## Abstract (precis)

**Summary:** We present a system for exposing mice to hypergravity and measuring the effects on their locomotion and activity. We include details of the design and implementation, and example experimental data.

## INTRODUCTION

Two of the most popular models for studying human or animal locomotion are the inverted pendulum for walking (Cavagna et al., 1976; Alexander, 1995) and the spring loaded inverted pendulum (SLIP) for running (Blickhan, 1989; McMahon and Cheng, 1990). In these models, the body is considered a point mass supported by a rigid (for walking) or compliant (for running) mass-less strut of length *L*, which is usually taken to be the effective leg length of the subject. A single traverse of the pendulum (i.e. half its period) represents the stance duration of the gait cycle during walking; the model for running adds compression and extension of the leg throughout stance, and introduces a ballistic aerial phase between stances. Although inverted pendulum-based models appear very simple, they have successfully been used to predict the speed at which the walk–run transition occurs (Usherwood, 2005; Hubel and Usherwood, 2013) to study motor control (Brenière and Do, 1991) and to design and control legged robots (Kajita et al., 2003). However, some aspects of locomotion are not easily explainable using these models; for example why small animals walk and run with proportionally longer stance times than larger animals (Alexander, 1984). Investigating the limitations of inverted pendulum models of locomotion may lead to advances in robotics and treatment of gait pathologies, as well as our fundamental understanding of legged locomotion.

The motion of an inverted pendulum can be described using the equation 
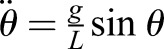
, where 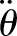 is angular acceleration, *g* is gravitational acceleration, *L* is length, and θ is angular displacement from the equilibrium point; testing the predictions of inverted pendulum-based models therefore requires the manipulation of either *L* or *g*. Differences in *L* are usually studied by comparing similar animals of different sizes (Gatesy and Biewener, 1991; Daley and Birn-Jeffery, 2018) or by comparing animals through ontogeny (Smith et al., 2010; Hubel and Usherwood, 2015; Usherwood et al., 2018); however both of these techniques may be influenced by confounding factors due to species differences or age-related musculoskeletal or neuromuscular changes. Some researchers have therefore opted to manipulate *g* by either reducing gravity (i.e. micro- or hypogravity) or increasing gravity (i.e. hypergravity). One of the oldest techniques for simulating reduced gravity for astronaut training has been to submerge the subject in a tank of water, using ballast to vary their buoyancy and hence their effective weight (Trout and Bruchey, 1969; Mirvis and Akin, 2011); the main disadvantage of this technique is hydrodynamic effects such as drag. A similar technique is to enclose the lower half of the subject's body in a pressurised chamber (Grabowski, 2010; Saxena and Granot, 2011), although this only exerts an upwards force on the portion of the body enclosed by the device.

The most commonly used technique to simulate hypogravity for research purposes has been to exert a constant upwards force on the subject as they run on a treadmill, either by using an actuator (Ivanenko et al., 2011) or a combination of pre-tuned springs and pulleys (Farley and McMahon, 1992; Donelan and Kram, 1997), resulting in a partial reduction in the subject's weight. This setup allows for more natural locomotion than neutral buoyancy systems; however the unloading force is only applied to the body and not the limbs, and care must be taken to minimize fluctuations in spring tension. A similar technique, using springs to exert a downwards force, has been used to study treadmill locomotion under simulated hypergravity (Chang et al., 2000; Lee et al., 2013).

True hypogravity (De Witt et al., 2010) and hypergravity (Cavagna et al., 2005) can be achieved when subjects walk or run on a treadmill during a parabolic flight. However, the change in gravity can only be maintained for short periods of time (typically <60 s), and the range of different gravity levels that can be achieved is limited.

Other researchers have used centrifuges to expose subjects to increased gravity; due to size limitations, human hypergravity experiments using this technique have been constrained to studies where limited movement is required [e.g. blood pressure (Petersson et al., 2006) or spatial orientation (Clark et al., 2015)]. However, the small size of rodents means that multiple animals can be housed in cages where they can move freely, meaning that as well as changes to muscle and bone properties, circadian rhythm and behaviour can also be studied. Table 1 gives an overview of the ranges of sizes and capacities of centrifuges that have been built by research groups around the world; many of these designs have been used in multiple studies since their construction.

**Table 1.**
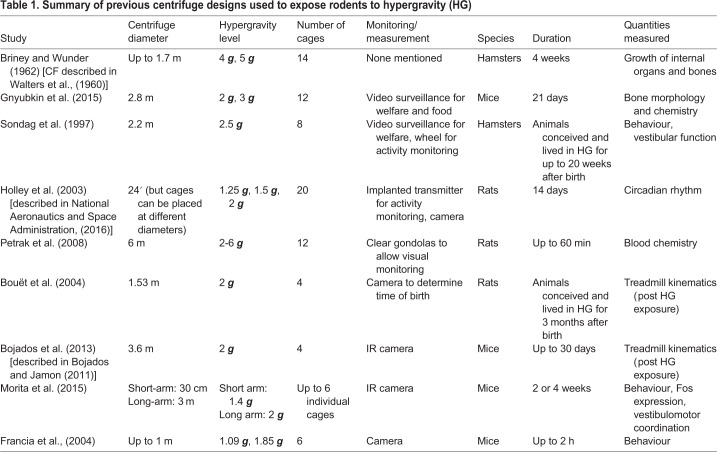
**Summary of previous centrifuge designs used to expose rodents to hypergravity (HG)**

A number of authors have studied the effect of hypergravity on locomotion and activity in mice, rats and hamsters. Sondag et al. (1997) investigated activity and gait in hamsters which had been born and reared in 2.5 ***g*** hypergravity; hamsters either remained at 2.5 ***g*** for 20 weeks or were removed from the centrifuge after 4 weeks and kept at 1 ***g*** for the remainder of the experiment. Daily wheel use was measured for each individual using a wheel outfitted with a bicycle computer. Subsequent to hypergravity exposure, gait parameters were recorded using video and a track with x-ray film that reacted to a chemical applied to the animals' feet. The hamsters exposed to hypergravity for 20 weeks had consistently low speeds and activity levels, whereas the control hamsters and those exposed for the shorter duration reduced their speeds and activity levels over time. The authors suggested that the hypergravity hamsters were just as motivated, but fatigued quicker. Hypergravity hamsters took smaller steps than controls, however there were no differences in duty factor (i.e. the proportion of stride for which a foot is in stance). Fuller et al. (2006) also used a wheel to measure activity, with the goal of comparing the effects of hypergravity and exercise on the soleus and plantaris muscles. Two groups of rats, one with access to wheels and one without, were exposed to 2 ***g*** for 60 days. There were also two control groups at 1 ***g***, with and without wheel access. Gross motor activity and wheel use was much lower in the 2 ***g*** rats; for the first 5 days the rats did not use the wheel at all, after which wheel use increased, but gross activity only reached 40% of the 1 ***g*** levels.

Bouët et al. (2004) studied rat locomotion in more depth, measuring kinematic parameters of rats which had been conceived, born and raised in 2 ***g***. A treadmill and an infrared tracking system (ELITE) were used to collect the locomotion data on the first day of adaptation to 1 ***g***. Compared to controls, the hypergravity rats had a high step frequency, lower stance and swing lengths, and lower duty factor. However, these effects were transient and disappeared within 28 days after exposure came to an end. Bojados et al. (2013) also studied treadmill kinematics in mice which had been exposed to 2 ***g*** at different stages of their development; one group was exposed from conception to 10 days after birth, one group was exposed from conception to 30 days after birth, and the third was exposed from 10 days after birth to 30 days after birth. At the age of 2 months, kinematics on a treadmill were recorded using high-speed video. Mice which were only exposed to 2 ***g*** for 10 days post birth (and therefore had not learned to walk in hypergravity) did not differ from controls, however the other two groups had higher stride frequency, shorter stance and stride length, and at walking (but not running) speeds they had lower duty factor (although only in the forelimb for mice exposed from conception to 30 days post birth). All mice exposed to hypergravity exhibited hyperextension of their legs, walking with a more upright posture.

**Fig. 1.**
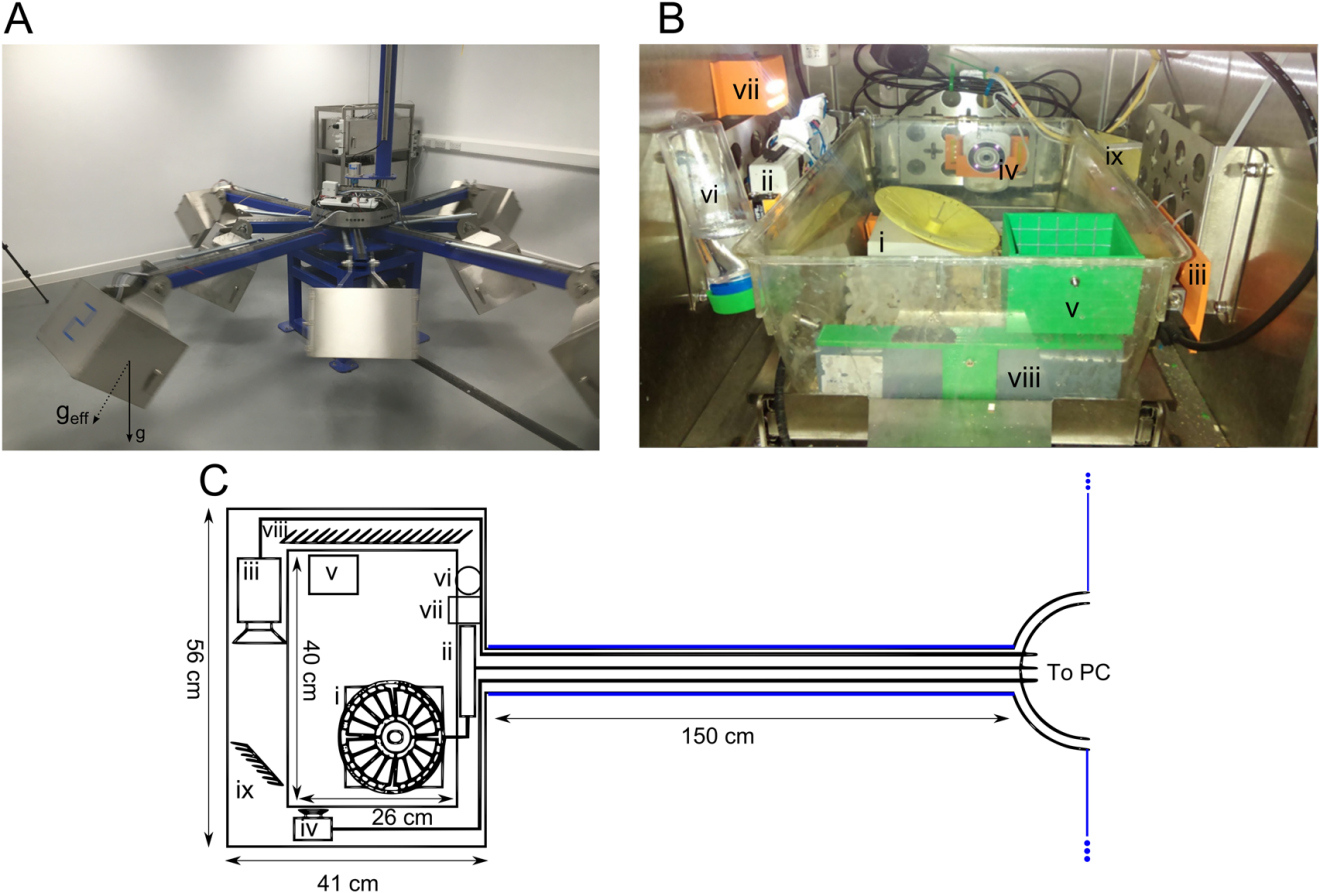
**Centrifuge structure and internal layout.** (A) Photo of the centrifuge in motion. The vector g denotes Earth's gravity, while the vector g_eff_ denotes the effective gravity within the gondola. (B,C) The interior of the gondolas: i, force-sensing exercise wheel; ii, USB-DAQ used to sample data from wheel and monitor temperature and humidity; iii, high-speed camera; iv, D-Link monitoring camera; v, food hopper; vi, water bottle; vii, timed LEDs; viii and ix, mirrors.

In this paper we present a centrifuge (shown in Fig. 1) for exposing small animals like mice to various levels of hypergravity and quantifying their behaviour and locomotion while in the hypergravity environment, rather than waiting until after exposure. As well as the centrifuge and integrated instrumentation, we discuss acclimatization and some results of exposing mice to varying levels of hypergravity.

## RESULTS AND DISCUSSION

### Comparison to previous systems

In terms of mechanical structure, the centrifuge presented here is comparable to those listed in Table 1. Its total diameter while rotating at full speed is approximately 4 m; larger than all the listed systems apart from those used by Petrak et al. (2008) and Holley et al., (2003). While diameter is limited largely by space and cost, a larger diameter reduces the influence of Coriolis forces, which may both disorient mice and affect their locomotion. However, the total capacity of this centrifuge is not as high as some other studies with up to 48 mice in eight cages. This was seen as sufficient for biomechanics studies, as each individual mouse will produce a high volume of strides. The main innovation of the system presented here is that measurements can be taken during hypergravity exposure, rather than limiting locomotion measurements until after the exposure. This has been enabled by the design and implementation of custom automated instruments, which do not require human interaction to record data, and can thus be used inside a rotating centrifuge.

### Example experimental data

The setup described in this paper was intended to fulfil two primary goals: (1) to demonstrate that we could expose mice to hypergravity at selected levels while maintaining good standards of welfare and (2) to measure activity, running speeds and gait parameters of mice undergoing hypergravity exposure.

**Fig. 2.**
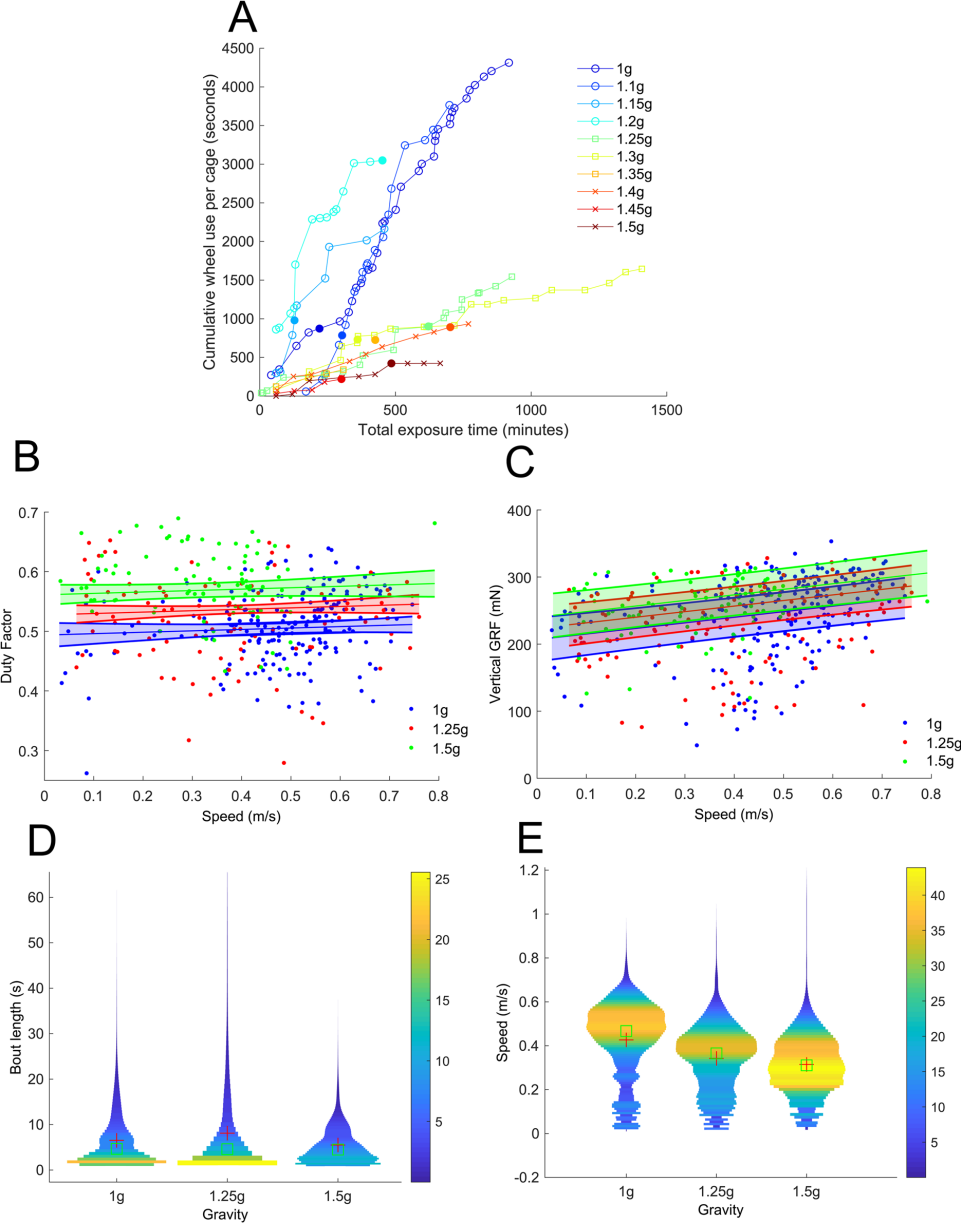
**Example locomotion data from mice exposed to a range of hypergravity levels.** (A) Cumulative running time versus cumulative exposure time per mouse. Different colours denote different gravity levels, while different markers denote number of mice. Empty circles denote all six mice, squares denote five mice and crosses denote two mice. Filled circles denote the points where mice were judged to have acclimatized to a gravity increment. (B) Changes in duty factor with speed at 1 ***g***, 1.25 ***g*** and 1.5 ***g***. (C) Changes in vertical GRF with speed at each gravity level. (D) Violin plots comparing the running bout durations at each gravity level. (E) Violin plots comparing the running speeds at each gravity level.

Goal 1 was achieved by exposing mice to increasing levels of hypergravity between 1.1 ***g*** and 1.5 ***g*** in 0.05 ***g*** steps. Fig. 2A shows how average wheel use per cage changed over the course of each acclimatization period. There was a slight but non-significant trend towards more exposure time being required for acclimatization at higher gravity levels [R^2^=0.269, *F*(8)=2.94, *P*=0.125], but there was no corresponding increase in wheel-use times until acclimatization [R^2^=0.12, *F*(8)=1.09, *P*=0.326]. This may indicate that wheel use under hypergravity is dependent more on general environmental acclimatisation, rather than training for increased exercise capacity.

Not all mice were able to acclimatize to every gravity level; three mice did not use the wheel at gravity levels higher than 1.35 ***g***, while one mouse developed a stereotypical circling behaviour 13 weeks after the start of the study and was euthanized on the advice of the Named Veterinary Surgeon (NVS). However, the FVB strain has been found to have a higher than average incidence of stereotypic behaviour (Nitezki et al., 2017) and the circling occurred both in the centrifuge (after which she was removed) and in the home cage, so it is possible that the stereotypic behaviour was not directly caused by the hypergravity exposure.

Goal 2 is illustrated in Fig. 2B–E. Data analysis was carried out using standard MATLAB functions and a significance level of *P*<0.05 was used. Fig. 2B shows example duty factor data (proportion of each stride where the foot is in contact with the ground) at different speeds at 1 ***g***, 1.25 ***g*** and 1.5 ***g*** based on kinematic data from the high-speed video camera, while Fig. 2C shows example vertical ground reaction force (GRF) data from the wheel at different speeds at 1 ***g***, 1.25 ***g*** and 1.5 ***g***. Likelihood ratio tests of the full linear mixed model (LMM) versus the model without the effect in question were used to calculate *P*-values for the effects of hypergravity and speed. Hypergravity caused significant increases in both duty factor (χ^2^=59.3, *P*<0.001) and GRF (χ^2^=18.6, *P*<0.001), although the increase in force was less than the increase in effective body weight. While speed caused a significant increase in GRF (χ^2^=26.0, *P*<0.001), it did not have a significant effect on duty factor (χ^2^=1.41, *P*=0.23).

Fig. 2D shows a violin plot of the bout durations selected by the mice as they ran at 1 ***g***, 1.25 ***g*** and 1.5 ***g***. A threshold of 1 s was applied to the data to remove small movements which did not represent running bouts. Shorter bouts were most common at all gravity levels, with a reduction in modal bout duration from 1.82 s at 1 ***g*** to 1.58 s at 1.25 ***g*** and 1.40 s at 1.5 ***g***. However, a Kruskal–Wallis one-way ANOVA showed that there was no significant difference between the bout durations at different gravity levels (χ^2^=1.24, *P*=0.54).

Fig. 2E shows a violin plot of the speeds selected by the mice as they ran at 1 ***g***, 1.25 ***g*** and 1.5 ***g***. A Kruskal–Wallis one-way ANOVA showed that there was a significant difference between the speeds at different gravity levels (χ^2^=406.7, *P*<0.001). Mean speed reduced from 0.43 m/s at 1 ***g***, to 0.34 m/s at 1.25 ***g*** and 0.31 m/s at 1.5 ***g***, while modal speed reduced from 0.49 m/s at 1 ***g***, to 0.39 m/s at 1.25 ***g*** and 0.29 m/s at 1.5 ***g***. However, the maximum speeds which the mice used increased slightly, from 0.87 m/s at 1 ***g***, to 0.89 m/s at 1.25 ***g*** and 1.07 m/s at 1.5 ***g***.

The system was successful in collecting large volumes of data at a range of gravity levels; on average 62 3-s videos were collected per session; this demonstrates that mice were still motivated to use the wheel even when exposed to hypergravity. This is similar to previous studies, which have also found that rodents are still motivated to use exercise wheels while exposed to hypergravity (Sondag et al., 1997; Fuller et al., 2006). As with the hamsters in Sondag et al. (1997), the mice also used lower mean and modal speeds at higher gravity levels. In studies which tested different levels of hypergravity such as Holley et al. (2003) and Francia et al., (2004), it was found that there was a dose effect, with animals taking longer to acclimatize to higher levels of hypergravity. We also measured a similar trend, although it was not statistically significant. Although this may be due to the lower gravity levels used in this study, it might also be that the dose effect was attenuated by our approach of exposing the mice to short periods of increasing levels of hypergravity, rather than a long duration at a high level, which allowed us to gather locomotion data within the first hour of exposure.

One disadvantage of using the wheel rather than a treadmill is that speed and bout length cannot be controlled by the experimenter; however, the mice were able to maintain similar speed ranges at all gravity levels, and had similar preferred bout lengths, meaning that over time sufficient data to compare gravity levels at different speeds can be obtained. Previous studies of the effects of hypergravity on rodent locomotion have only used one or two speeds, typically on the mid–low end of the speed range [e.g. 33 cm/s and 50 cm/s for Bojados et al. (2013) and 21 cm/s and 33 cm/s for Bouët et al. (2004)], rather than a range of different speeds. Furthermore, the data in these papers was collected after the animals were removed from hypergravity after a long period of exposure; it therefore represents animals adapting to a lower gravity than they are used to, rather than animals adapting to higher levels of gravity as presented here. Despite these differences, there are some similarities in the trends in the data presented here with the data from previous studies. Mice (Bojados et al., 2013) and rats (Bouët et al., 2004), both exhibited lower duty factors (to an extent) when walking at gravity levels lower than they were used to (i.e. at 1 ***g***), while we measured higher duty factors at higher gravity levels. Similarly, limb hyperextension (i.e. walking with more upright limbs) was observed in mice (Bojados et al., 2013) and hamsters (Sondag et al., 1997) undergoing effective lower gravity, while our measurements of increased duty factor and lower increase in GRF than increase in effective body weight point to more crouched, compliant legs in mice undergoing exposure to increased gravity.

The automatic selection and synchronization tool was able to distinguish the videos that would be suitable for analysis. However, the automated nature of the data collection meant that there was still more ‘noise’ in the set of videos than there might be in a manually triggered dataset, for example: two mice might run on the wheel at the same time, so different individuals might be in frame at different points in the same video; or a mouse might stop running momentarily and coast on the still-moving wheel. This limits the extent to which the data analysis can be automated without implementing more complex machine vision algorithms.

Due to the length of time that acclimatization took, and concerns about the initially unknown effects of hypergravity on welfare, we did not expose mice to hypergravity levels as high as some other studies, and also exposed mice to hypergravity for shorter periods of time. The highest gravity in this paper is 1.5 ***g***, versus 5 ***g*** as used previously (Briney and Wunder, 1962), and sessions were 1 h each, versus 3 months (Bouët et al., 2004). However, welfare does not appear to have been negatively impacted – mice continued to engage in voluntary locomotion and maintained their body condition to the satisfaction of experienced animal technicians and the NVS. As the centrifuge is mechanically capable of achieving hypergravity levels up to 10 ***g***, and suitable environmental conditions can be achieved within the gondolas, further experiments of longer term and higher gravity level are feasible in terms of both practicality and – with appropriate continued monitoring – animal welfare. The results of this study can be used as a starting point for planning further experiments, in terms of estimating how much exposure time to allocate for acclimatization, and how much is likely to be required to gather the appropriate amount of data.

## MATERIALS AND METHODS

### Centrifuge design

Fig. 1A shows a photo of the centrifuge; it is comprised of four 150 cm arms and four 125 cm arms attached to a central platform, 60 cm in diameter. It is driven using a 1.5 kW motor (TEC Electric Motors, Droitwich, UK), and is capable of achieving a speed of up to 60 rotations/min (1 Hz). Each arm carries a ‘gondola’ – an enclosed container which can swing freely between 90° and 0° to the axis of the arm. When the centrifuge is stationary, the z-axis of the gondolas (i.e. the cage floor-lid axis) is perpendicular to the arms. As the speed increases, the gondolas swing out until, at the highest speed, the z-axis is close to parallel with the arms. This ensures that the effective gravity vector always passes approximately perpendicular to the floors of the cages. A dashpot on each arm damps the motion of the gondolas to smooth oscillations of the gondola-arm angle. The level of effective gravity that the mice experience (*g_eff_*) is the resultant of Earth's gravity field *g*, and the centrifugal acceleration *ω*^2^(*r*_1_+*r*_2_sin*θ*), where *ω* is speed of rotation, *r_1_* is the distance from the motor pivot to the gondola pivot, *r_2_* is the distance from the gondola pivot to the floor of the cage, and *θ* is the angle of the cage to the vertical. Effective gravity is thus 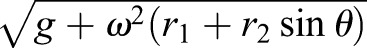; at the maximum speed of 60 rpm, this means that the effective gravity within the gondolas on the 150 cm arms is 10 times Earth's gravity, or 10 ***g***, and the effective gravity within the gondolas on the 125 cm arms is 6 ***g***.

A gantry arm is connected to the central platform via a slip ring with 240 V 10 A AC, Ethernet and gas connectivity. This allows power to be supplied, data to be transmitted to and from the centrifuge, and oxygen and CO_2_ concentrations in the gondolas to be measured. Semi-enclosed conduits run along each of the arms, carrying power, data and gas lines from the central platform to the gondolas. The gondolas each hold a standard 1291H rodent cage (Tecniplast UK, London, UK), which can house up to six mice. The cage sits tightly in a tray, which is on rollers for ease of access, and there is an access door at each end of the gondola. The top, sides and doors of the gondola have frames, which are used to mount instruments and secure cables, while the base of the gondola is perforated to allow ventilation. LED lights mounted inside the gondola can be turned on and off manually, or can be programmed to automatically produce a desired day–night cycle.

The centrifuge is housed in a purpose-built room which can maintain the required environmental conditions within the gondolas, as recommended by the Home Office (UK Home Office, 2014): temperature is kept between 19°C and 23°C, humidity is kept between 45% and 65%, and the ventilation system can achieve 15 changes of air/h. A door-interlocking system prevents access to the room while the centrifuge is in motion or the centrifuge being turned on while the door is unlocked; this is necessary since while the centrifuge was rotating at full speed the gondolas are travelling at approximately 12.6 m/s, and there is limited space around the centrifuge to avoid being hit. The corners of the room are also kept clear to act as emergency refuges, each with centrifuge stop buttons; and centrifuge start-up takes place over a number of minutes.

### Instrumentation and cage setup

Fig. 1B and C show the layout of the gondolas. All measurements and recordings take place autonomously so that experiments can be conducted overnight or for long periods of time. Two instruments were selected for studying locomotion: an exercise wheel capable of measuring running speed and vertical GRF, and a high-speed camera, which is used to collect data on foot contact times and posture.

The 3D-printed force-sensing exercise wheel is described in more detail in Smith et al. (2015): the rotating part of the wheel consists of 16 deflectable pads, with a magnet set into the underside of each one. An array of Hall sensors in the base of the wheel measures changes in the magnetic field, which can then be used to calculate vertical ground reaction force, speed and running bout duration. When the wheel is moved, a NI USB-6210 DAQ (National Instruments Corporation, Austin, USA) begins logging data from the wheel at 3 kHz, while custom LabVIEW (National Instruments Corporation) code also triggers high-speed video capture at a rate of up to 200 Hz for 3 s, using the Basler acA2000-165umNIR camera (Basler AG, Ahrensburg, Germany). This camera is capable of imaging using both visible and infrared light, meaning that it can be used during the dark phase when mice are typically most active. As well as collecting these kinematic and kinetic data, the LabVIEW code also samples once 1 h from a Microchip MCP9700AT-E/LT temperature sensor (Microchip Technology inc., Chandler, USA), and a Honeywell HIH-5031-001 humidity sensor (Honeywell International Inc., Morris Plains, USA) inside the gondola. Since there is limited data available on the welfare effects of hypergravity in mice, a D-link DCS-932L infrared security camera (D-Link, Taipei, Taiwan) is used to observe behaviour in real time, and is also motion-triggered to record activity. Plans for the wheel, and all data collection software can be accessed online at https://www.rvc.ac.uk/static/sml/smith_usherwood_centrifuge_code_2019.zip.

### Data processing

One of the challenges of collecting continuous data over a long period relative to the sample rate (as is the case for high-speed video and the wheel for long running bouts) is that it can take a significant period of time to write the data from the buffer to the hard drive, during which more data cannot be collected. In order to minimize turn-around time, the wheel and video data were saved as raw binary files; custom MATLAB and C# code were later used to batch process and synchronize the data. Triggering data collection based on wheel movement resulted in a large number of data files; however, not all of these were suitable for analysis, either because the mouse jumped off the wheel immediately after jumping on, or because an object (such as another mouse or some bedding) occluded the camera. The first step was therefore to identify which files would be suitable for further processing. This was achieved using a custom MATLAB script which linked each video file with the corresponding wheel data file. It then calculated the mean speed of the bout to determine if the mouse was actually running, and the proportion of white pixels in the video to determine if a mouse was visible in the image. If the camera was occluded, there was a low number of white pixels per frame, but if there was a clear shot of a running mouse there was a high number of white pixels per frame. Once these files had been identified, they were converted to .avi files using a custom C# program, and digitized using Kinovea (https://www.kinovea.org, accessed 22/01/2019). Custom MATLAB code (available in the Data availability section) was then used to synchronise the kinematic data from the videos with speed and force data from the wheel. All data processing software can be accessed online at https://www.rvc.ac.uk/static/sml/smith_usherwood_centrifuge_code_2019.zip.

### Acclimatization and data collection

All procedures were carried out under an approved Home Office project license (number PPL 70/8281) and in accordance with the Animals (Scientific Procedures) Act, 1986 (UK) (amended 2013).

The initial experimental cohort consisted of six female FVB mice (*Mus musculus* Linnaeus) sourced from Charles River UK Ltd and aged 9 weeks at the start of the study. Each mouse was marked with a number of lines on its tail with a Sharpie pen to identify it for husbandry purposes. However, since all the mice arrived from the same source at the same time, and were the same age and sex, for the purpose of data analysis we assumed that any differences between individuals were insignificant compared to differences due to hypergravity exposure. Mice were first acclimatized to the centrifuge before exposure to hypergravity; three mice were placed in a gondola with a small amount of bedding from the home cage to reduce stress, and were allowed to explore and use the wheels for a period of 2 h. Food and water were freely available. This was repeated on two consecutive days. The procedure for exposure to hypergravity was similar; three mice were placed in a gondola on one of the 150 cm arms, with food, water and nesting material. The centrifuge was slowly accelerated to the desired speed over a period of 156–318 s, and this speed was maintained for 1 h. During the constant-speed phase, speed and force data was logged at 3 kHz, and video was recorded at 120 fps when the mice ran on the wheel; no data was recorded in the acceleration and deceleration phases. The centrifuge was then decelerated slowly, and the mice returned to their home cage for at least 1 h between hypergravity sessions. This was carried out for gravity levels between 1.1 ***g*** and 1.5 ***g***, in increments of 0.05 ***g***. Mice were acclimatized to each gravity level before being exposed to the next and this was considered to have occurred when the wheel was triggered at least 50 times/h-long session for two consecutive sessions, and the mice had been observed eating and drinking at that hypergravity level. During acclimatization, an experimenter observed the mice via the D-Link camera, so that the experiment could be ended if the mice appeared distressed. The mice were also weighed weekly as a welfare measure.
